# *In silico* exploration of biosynthetic gene clusters in marine *Streptomyces* sp. and *Nocardiopsis* sp. from the western coast of India: Genome-based profiling using whole genome sequencing

**DOI:** 10.1016/j.jgeb.2025.100483

**Published:** 2025-03-25

**Authors:** Hithesh Kumar, Santhiya Vijayakumar, Neha Shintre, Vaijayanti Tamhane, Neelima Deshpande, Tushar Joshi, Shalini Mathpal, Anand Anbarasu, Sudha Ramaiah

**Affiliations:** a Medical and Biological Computing Laboratory, School of Biosciences and Technology (SBST), Vellore Institute of Technology (VIT), Vellore 632014 Tamil Nadu, India; b Department of Bio-Sciences, School of Biosciences and Technology (SBST), Vellore Institute of Technology (VIT), Vellore 632014 Tamil Nadu, India; c Content Writer - Biology, JoVE, India; d Institute of Bioinformatics & Biotechnology, Department of Biotechnology, Savitribai Phule Pune University, Pune, Maharashtra, India; e Abasaheb Garware College, Pune, Maharashtra, India; f Department of Biotechnology, School of Biosciences and Technology (SBST), Vellore Institute of Technology (VIT), Vellore 632014 Tamil Nadu, India

**Keywords:** Actinobacteria, Secondary metabolites, Genome sequencing, Bioactive compounds, Biosynthetic gene clusters

## Abstract

*Actinomycetes* are known for their ability to produce bioactive compounds with significant potency of antibiotics and natural product synthesis. With the growing threat of antimicrobial resistance, effective treatment for many infections has become increasingly challenging. Our study aims to explore the secondary metabolites produced by *Actinomycetes* isolated from marine sponge samples collected from the west coast of India using *in silico* approaches. The pre-processed high-throughput Illumina sequencing reads from six *Actinomycete* genomes showed high quality. Initial BLAST analysis followed by phylogenetic analysis revealed that isolates A01 and A96 closely matched *Nocardiopsis* sp., while isolates A03, A45, A57, and A90 were closely related to *Streptomyces* sp. *In silico* biosynthetic gene clusters (BGC) prediction indicated that *Streptomyces* sp. A57 had the highest number of BGCs, with 28 clusters identified. All *Streptomyces* sp. (A03, A45, A57, and A90) were predicted to contain a high number of terpene gene clusters. Ectoine was commonly found in all genomes of *Streptomyces* sp. and *Nocardiopsis* sp. Most of the BGCs identified in *Actinomycete* genomes revealed less similarity to the known BGCs, indicating their potential for producing novel secondary metabolites. The study reveals the genomic potential of the *Actinomycetes* by providing new insights into the ecological roles and potential applications of marine *Actinomycetes*, highlighting their promise as candidates for the discovery of new pharmaceuticals. Future investigations could benefit from integrating functional genomics and metabolomics to gain deeper insights into the metabolic pathways governing the biosynthesis of these secondary metabolites.

## Highlights

• *Actinomycetes* produce secondary metabolites that exhibit antimicrobial activity.
• *Streptomyces* sp. A57 showed the highest BGC clusters among other studied samples.
• Less similarity of the BGC clusters reveal the novel secondary metabolite potential.
• Novel allelic profiles were exhibited by all the *Streptomyces* samples.

## 1 Introduction

Antimicrobial resistance (AMR) has become a global public health concern, and it poses great difficulty in treating microbial infections. As the emergence of AMR pathogens increased over the years, the use of antibiotics also substantially increased causing a shortage of antibiotics as an additional threat to the AMR crisis. This indicates the urgent need to explore novel biomolecules as a source of drug molecules to fight against multidrug-resistant infections.

In recent years, *Streptomyces* has gained enormous research attention due to its prolific secondary metabolite production exhibiting a wide range of pharmaceutical activities such as antimicrobial, antifungal, antiviral, anticancer, anti-angiogenesis, antioxidant, antidiabetic, cytotoxic, immunosuppressive and other relevant biological activity.^1^ *Streptomyces* is the largest and most widespread genus of the phylum actinobacteria in both terrestrial and marine environments in terms of secondary metabolite production^2^. It exhibits a wide range of metabolic and physiological characteristics as the organism adapts to extreme marine environment conditions such as varying temperature, low pH, high pressure and salt concentrations, and low oxygen and nutrient content.^3^

*Nocardiopsis* is another abundant genus in marine environments that comes under *Actinomycetes*.^4^ These genera distributed across numerous environmental conditions are a source of natural products in both marine and terrestrial ecosystems.^5^ These organisms survive even in harsh environments due to their versatile genetic composition, enzyme production, and production of surfactants and solutes.^5^, ^6^ The bacterial adaptation to extreme environmental conditions gives rise to the production of secondary metabolites unique to that habitat, which act as the source for novel drugs to treat human diseases.^7^

The biosynthesis of secondary metabolites is clustered in a repetitive region of the genome termed biosynthetic gene clusters (BGCs).^8^ The majority of these gene clusters encode for non-ribosomal peptide synthases (NRPS), polyketides, lantipeptides, terpenes, siderophores, bacteriocins, and other ketide synthases.^9^ Whole genome sequencing (WGS) results of *Streptomyces* species revealed that each species encodes for 25–50 BGCs, but most of them remain “silent” or “cryptic” under laboratory culture conditions.^10^ Rapid advancement in deep-sea ocean technology and genomic sequencing has enabled researchers to delve deeper into the genomes of microorganisms capable of producing distinct secondary metabolites that have accelerated the discovery of new drugs in the pharmaceutical industry.^11^ In this study, we examined high-quality genomic sequences of *Nocardiopsis* sp. and *Streptomyces* sp. isolated from the western coast of the India to investigate their secondary metabolite profiles. Our findings underscore the potential of these metabolites in the discovery of novel antibiotics.

## 2 Materials and methods

### 2.1 Data collection and sequencing

The samples were collected from different locations on the west coast of India, namely Harnai, Ade, and Goa. The sample type and the location of the collected marine samples are provided in Table 1**.** Small sponge tissue samples were collected without harming the colonies or habitat. Samples were rinsed in sterile Poor Ravan Saline (PRS) broth^12^ to remove debris and were stored in sterile tubes with the same medium. Sediment samples near the sponge were collected in sterile tubes. All samples were transported on ice and processed fresh for microbial isolation.

**Table 1:** Sample type and locations of the collected marine samples.

Isolate number	Sample type	Location	GPS Co-ordinates	Genus
A01	Sponge	Harnai	17°48′29.6″N, 73°05′47.1″E	*Nocardiopsis* sp.
A96	Sediment	Ade	17°52′19.4″N, 73°04′36.8″E	*Nocardiopsis* sp.
A03	Sediment	Ade	17°52′19.4″N, 73°04′36.8″E	*Streptomyces* sp.
A45	Sediment	Harnai	17°48′29.6″N, 73°05′47.1″E	*Streptomyces* sp.
A57	Sponge	Ade	17°52′19.4″N, 73°04′36.8″E	*Streptomyces* sp.
A90	Sediment	Goa	15°21′08.4″N, 73°46′41.8″E	*Streptomyces* sp.

To reduce non-sporulating bacteria, samples were heat-treated at 60 °C for 15 min. Sponge samples were homogenized in PRS medium and serially diluted up to 10^−5^ Sediment samples were shaken vigorously and similarly diluted. Aliquots (0.1 ml) of undiluted, 10^−3^, and 10^−5^ dilutions were spread-plated in triplicate on Zobell Marine Agar^13^. Nalidixic acid and cycloheximide (25 μg/ml) were added to inhibit gram-negative bacteria and fungi.^14^ Plates were incubated at 30 °C and monitored daily for 21 days. Colonies resembling actinobacteria were isolated, subcultured for purity, and stored on diluted Zobell marine agar at 4 °C for further genome sequencing. Genomic DNA was extracted from the cultured samples (A01, A03, A45, A57, A90, and A96) and processed for 16S rRNA identification, followed by Whole Genome Sequencing (WGS). WGS paired-end libraries were prepared for all extracted samples for Illumina sequencing. Genomic sequencing was conducted at Agrigenome, Kerala, India, using the Novaseq 6000 Illumina platform. The raw genomic sequences, along with accompanying metadata, have been submitted to the National Center for Biotechnology Information (NCBI) under the bioproject accession number PRJNA1042969.

### 2.2 Data quality check and pre-processing

The raw genomic paired-end datasets underwent data QC processing using the FastQC toolkit to assess their quality. Subsequently, the QC reports for all datasets were compiled using the MultiQC toolkit.^15^, ^16^ Sequencing data were trimmed for Illumina universal adapters, sequencing library adapters, redundant sequences, and low-quality bases using Trimmomatic v0.36, with a quality cut-off value of Q30 (Phred Quality Score).^17^ Additionally, low-quality reads and short sequences with minimal lengths of less than 50 base pairs (bp) were trimmed to maintain sequence quality. Read statistics for both raw and pre-processed reads were generated using the Seqkit toolkit.^18^

### 2.3 *De novo* genome assembly and quality assessment

*De novo* genome assembly of all samples was generated using high-quality pre-processed reads with the St. Petersburg genome assembler (SPAdes) v3.15.2.^19^ The SPAdes assembler performed various steps, including constructing a de Bruijn graph, error and artifact correction, and scaffolding in the genome assembly pipeline. The data input type considered for the assembly was paired-end reads. An in-house developed Python script was used to generate genome assembly statistics. Additionally, GC content and other genome statistics were calculated using Seqkit v0.16.1.^18^ The quality of the genomes was assessed using quantitative measurements of genome assembly completeness relative to evolutionary relationship data of Benchmarking Universal Single-Copy Orthologs (BUSCO) v5.1.2.32.^20^ The assembled genomes, along with their corresponding metadata, were also submitted to the National Center for Biotechnology Information (NCBI) under the Bioproject PRJNA1042969.

### 2.4 Genome annotation and secondary metabolite analysis

Gene prediction and genome annotation of the assembled genomes were processed using Prokka v1.12.^21^ This tool predicts the coordinates of candidate genes, tRNA (Transfer ribonucleic acid), rRNA (Ribosomal ribonucleic acid), and ncRNAs (non-coding RNAs). The predicted genes were functionally annotated using COGclassifier v1.0.5 to categorize them into distinct Clusters of Orthologous Genes (COGs). The assembled genomes of *Streptomyces* sp. and *Nocardiopsis* sp. were evaluated for their secondary metabolites and biosynthetic gene clusters using the online tool antiSMASH bacterial version 7.0 (accessed on 05–12-2023).^22^ The detection strictness parameter was set to high to detect well-defined clusters comprising all the vital parts. Visual representations illustrating the distribution of different coding sequences (CDS) and BGCs throughout the genome were created using DNA plotter.^23^

### 2.5 Multiple sequence alignment and phylogenetic analysis

Multiple sequence alignment of the 16S rRNA sequences from all *Actinomycetes*, along with other reference sequences from valid *Streptomyces* and *Nocardiopsis* species (Top hits from BLAST against NR database with duplicates removed), was conducted using MAFFT v7.490, with a maximum of 1000 iterations.^24^ Following the alignment, a maximum-likelihood phylogenetic tree was constructed using IQ-Tree v1.6.12 software, including an ultrafast bootstrap analysis with 1000 replicates to assess the robustness of the tree topology.^25^ The best-fit model was selected based on the Bayesian Information Criterion (BIC). The resulting phylogenetic tree was visualized using Interactive Tree of Life (iTOL) v6.^26^, ^27^, ^28^

Although the 16S rRNA gene has been widely used for bacterial classification, its high genetic similarity among closely related species limits its discrimination potential.^29^ To overcome this limitation, we also constructed phylogenetic trees using concatenated housekeeping genes, which evolve at a faster rate and provide better resolution for species differentiation.^30^, ^31^ For *Streptomyces* species, the genes *atpD, gyrB, recA, rpoB,* and *trpB* were considered, while for *Nocardiopsis* species, *gyrB, rpoB*, and *sodA* were used.^32^, ^33^ Multiple sequence alignment was performed using MAFFT v7.940 with 1000 iterations. Phylogenetic trees were constructed using IQ-TREE v1.6.12 with 1000 bootstrap replicates and visualized using iTOL v6.

In addition, the average nucleotide identity (ANI) of *Nocardiopsis* sp. (A01, and A96) and *Streptomyces* sp. (A03, A45, A57, and A90) along with their top corresponding reference genomes identified through BLAST analysis was calculated using OrthoANI tool.^34^

### 2.6 Multilocus sequence typing (MLST) analysis

The MLST analysis was performed for the four isolates of *Streptomyces* sp. Subsequently, we identified sequence types (STs) using allelic profiles consisting of six housekeeping genes (*atpD, gyrB, recA, rpoB, and trpB*) using MLST v2.0 webserver, managed by the Center for Genomic Epidemiology at the Technical University of Denmark.^35^

## 3 Results

### 3.1 Quality assessment of sequencing data

Initially, 16S rRNA identification was performed using NCBI BLAST (blastn) analysis obtained via PCR. Based on the 16S rRNA results, four samples were identified as *Streptomyces* sp. (A03, A45, A57, and A90), and two samples were identified as *Nocardiopsis* sp. (A01 and A96). The raw genomic reads after trimming low-quality sequences and quality control yielded high-quality paired-end reads of 423,628,838 for A01, 338,510,006 for A03, 397,126,698 for A45, 369,996,676 for A57, 329,757,794 for A90, and 409,754,404 for A96. The quality assessment also indicated that the reads have high overall accuracy, with a Phred score of Q30 or higher, ranging between 93.9 % to 95.2 % **(Table S1a-b and Fig. S1).**

### 3.2 Genome assembly and annotation

The genomes of all samples were subjected to *de novo* assembly using SPAdes. The genome size of the four *Streptomyces* sp. strains (A03, A45, A57, and A90) ranged from 4.92 Mbp to 5.88 Mbp. The average genomic GC content of these *Streptomyces* sp. strains was 69.34 %. The N50 values for the genomes ranged from 184,991 to 471507 bp. Quality assessment using BUSCO with the Streptomycetales_odb10 database showed more than 99.4 % completeness with single-copy genes, indicating high-quality assemblies for *Streptomyces* sp. strains **(Fig. S2).** The number of coding sequences predicted through Prokka ranged from 6569 to 7111. More details on gene prediction are provided in **Table S1c.**

**Fig. 1:**
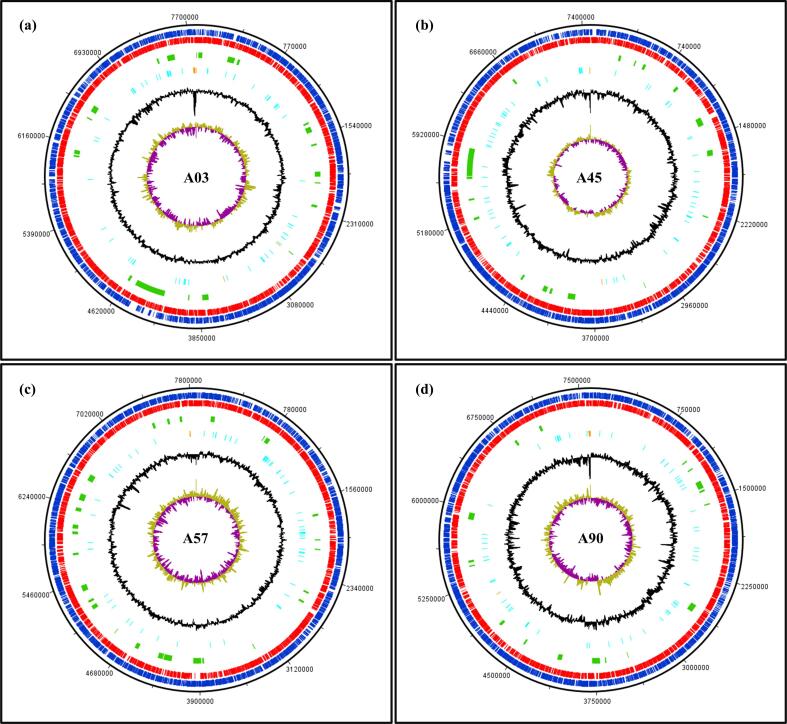
Circular genome maps of the chromosomes of *Streptomyces* sp. isolates (a) A03, (b) A45, (c) A57, and (d) A90 generated using DNA Plotter v18.2.0. Each circle, from outermost to innermost, represents coding sequences (CDS) on the positive strands (blue) and negative strands (red), putative BGCs regions (green), tRNA and rRNA genes (in cyan and orange, respectively), GC percentage plot (black), and GC skew (olive and purple). (For interpretation of the references to colour in this figure legend, the reader is referred to the web version of this article.)

**Fig. 2:**
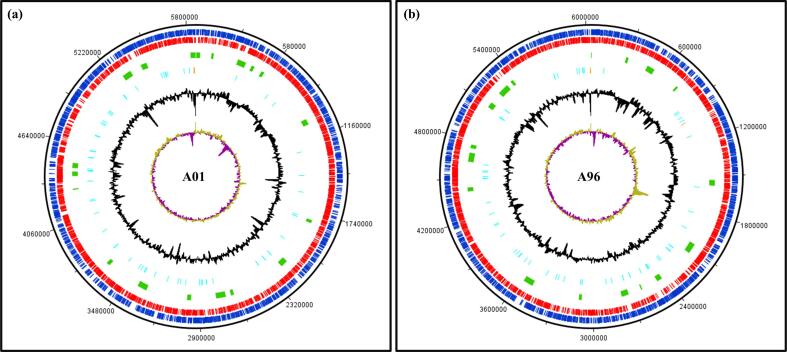
Circular genome maps of the chromosomes of *Nocardiopsis* sp. isolates (a) A01 and (b) A96 generated using DNA Plotter v18.2.0. Each circle, from outermost to innermost, represents coding sequences (CDS) on the positive strands (blue) and negative strands (red), putative BGCs regions (green), tRNA and rRNA genes (in cyan and orange, respectively), GC percentage plot (black), and GC skew (olive and purple). (For interpretation of the references to colour in this figure legend, the reader is referred to the web version of this article.)

The genome size of *Nocardiopsis* sp. A01 and A96 were 6.28 Mbp and 6.06 Mbp, respectively, with an average GC content of 69.71 %. The N50 values were 692,549 and 617594 bp for *Nocardiopsis* sp. A01 and *Nocardiopsis* sp. A96 respectively. The quality of both samples was high, as the assemblies were nearly fully complete, assessed using Streptosporangiales_odb10 as a reference database. A total of 5096 and 5289 coding sequences were predicted for *Nocardiopsis* sp. A01 and *Nocardiopsis* sp. A96, respectively (Fig. 1, Fig. 2).

The coding sequences (CDS) assigned to clusters of orthologous groups (COG) functional categories ranged from 5,187 to 5,465 among *Streptomyces* sp. strains A03, A45, A57, and A90. *Streptomyces* sp. A03 exhibited the highest proportion of genes classified into functional categories, with 5,465 CDS (79.68 %). The COG functional annotation revealed that all strains of *Streptomyces* sp. displayed a high number of genes associated with Transcription (COG K), carbohydrate transport and metabolism (COG G), and signal transduction mechanisms (COG T). Additionally, *Nocardiopsis* sp. A01 and A96 had 4, 021 and 4,137 CDS classified into COG functional categories, respectively. In both *Nocardiopsis* starins, the highest number of genes were classified for transcription (COG K) and amino acid transport and metabolism (COG E) **Table S2.**

### 3.3 Phylogenetic analysis of *Actinomycete* isolates

**Fig. 3:**
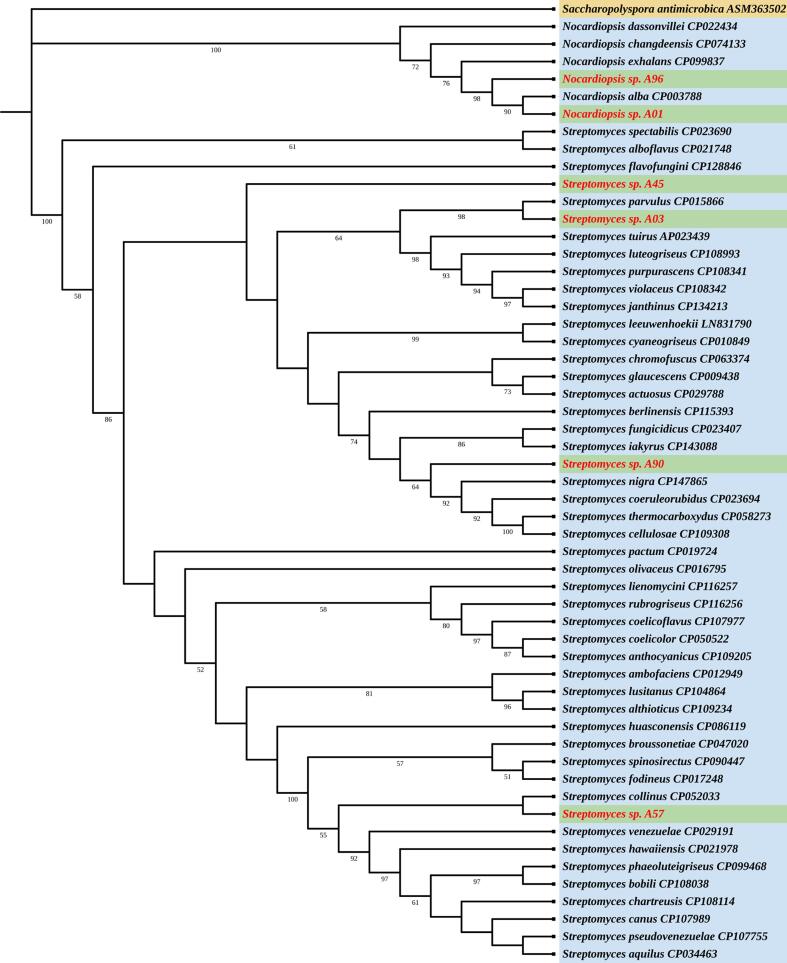
Phylogenetic relationship among the 16S rRNA sequences of *Streptomyces* sp. (A03, A45, A57, and A90) and *Nocardiopsis* sp. (A01 and A96) isolates with other valid *Streptomyces* and *Nocardiopsis* species. *Saccharopolyspora antimicrobica* used as an outgroup.

Phylogenetic analysis based on 16S rRNA sequences from *Streptomyces* sp. and *Nocardiopsis* sp. isolates, along with sequences from other *Streptomyces* and *Nocardiopsis* species, revealed their evolutionary relationships. The NCBI BLAST analysis results of 16S rRNA sequences against the NR database for phylogenetic analysis are provided in **Table S3.** The phylogenetic tree of the 16S rRNA gene branched distinctly for *Streptomyces* species, *Nocardiopsis* species and outgroup. The tree showed that *Nocardiopsis* sp. A01 clustered with *Nocardiopsis alba* ATCC BAA-2165, both of which formed a clade with *Nocardiopsis* sp. A96, indicating close genetic similarity. Similarly, *Streptomyces* sp. A57 was closely related to *Streptomyces collinus* strain L2, as they clustered together in a single clade. Additionally, *Streptomyces* sp. A03 clustered with *Streptomyces parvulus* strain 2297, suggesting a close evolutionary relationship. *Streptomyces* sp. A45 and *Streptomyces* sp. A90 did not cluster with any of the other *Streptomyces* species (Fig. 3).

**Fig. 4:**
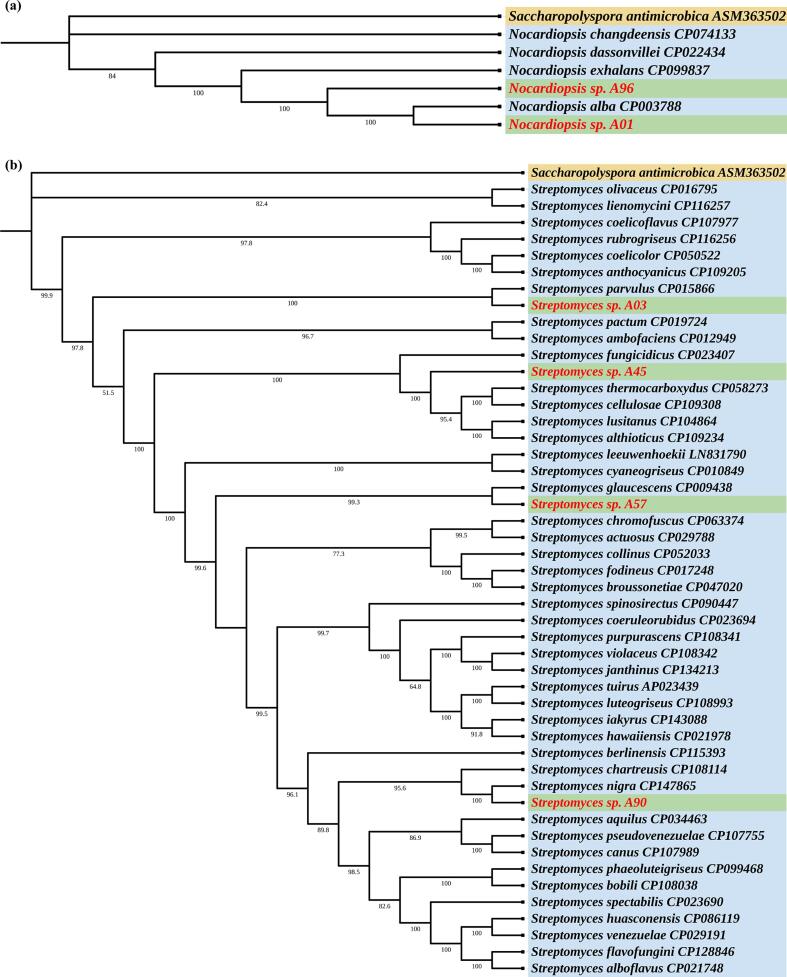
Phylogenetic tree based on housekeeping genes: (a) *gyrB, sodA, and rpoB* of *Nocardiopsis* sp. (A01 and A96) and other reference *Nocardiopsis* species (b) *atpD, trpB, rpoB, recA, and gyrB* of *Streptomyces* sp. (A03, A45, A57, and A90) and other reference *Streptomyces* species.

The phylogenetic tree of concatenated housekeeping gene sequences of *Nocardiopsis* sp. A01 and A96 displayed similar clustering as in the 16S rRNA phylogenetic tree. In the case of *Streptomyces* species, the phylogenetic analysis revealed that *Streptomyces* sp. A03 was closely related to *Streptomyces parvulus* strain 2297, while *Streptomyces* sp. A57 showed a close genetic similarity with *Streptomyces glaucescens* strain GLA.O. Additionally, *Streptomyces* sp. A90 clustered with *Streptomyces nigra* strain LM01, further revealing its close evolutionary relationship (Fig. 4**a and 4b**).

Among the *Streptomyces* strains, *Streptomyces* sp. A03 exhibited the highest ANI value 99.25 % with *Streptomyces parvulus* strain 2297. Notably, *Streptomyces* sp. A90 shared a very ANI value (92.50 %) with *Streptomyces nigra* strain LM01. Additionally, *Streptomyces* sp. A45 (83.44 %) and *Streptomyces* sp. A57 (83.47 %) displayed a close similarity with *Streptomyces coeruleorubidus* strain ATCC 13740 **(Fig. S3a).** For the *Nocardiopsis* samples, both *Nocardiopsis* sp. A01 and *Nocardiopsis* sp. A96 displayed high sequence similarity to *Nocardiopsis* alba ATCC BAA-2165, with ANI values of 99.18 % and 96.33 %, respectively. Interestingly, *Nocardiopsis* sp. A01 and A96 also shared a high sequence similarity (ANI value 96.39 %) **(Fig. S3b)**.

### 3.4 Genome mining

**Fig. 5:**
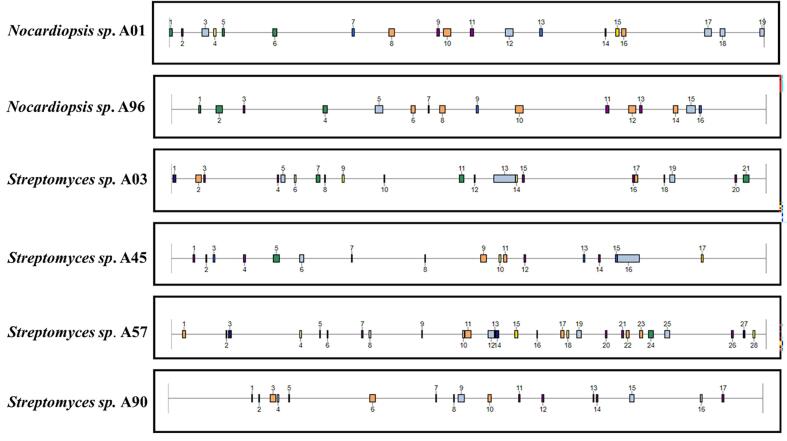
Visual representation of total number of BGCs across all isolates including *Streptomyces* sp. and *Nocardiopsis* sp.

An analysis using antiSMASH revealed that among the investigated isolates, *Streptomyces* sp. A57 encoded the highest number of BGCs (28 BGCs), followed by *Streptomyces* sp. A03 (21 BGCs). Both *Streptomyces* sp. A45 and *Streptomyces* sp. A90 harbored 17 BGCs each (Fig. 5). *Nocardiopsis* sp. A01 possessed 19 BGCs, while the least BGCs were identified in *Nocardiopsis* sp. A96 (Table 2).

**Table 2:** Biosynthetic gene clusters of *Nocardiopsis* sp. and *Streptomyces* sp. using antiSMASH tool.

**Biosynthetic compounds**	**A01**	**A03**	**A45**	**A57**	**A90**	**A96**
terpene	2	5	4	4	4	2
lanthipeptide-class-i	1	1	1	2	−	−
CDPS	1	−	−	−	−	1
NRPS	1	2	−	−	−	1
lassopeptide	1	−	3	−	−	2
T1PKS	2	−	−	1	−	4
T2PKS	1	1	1	−	2	1
betalactone	1	−	−	−	−	1
ectoine	1	1	1	2	1	1
thiopeptide	1	−	−	1	−	−
indole	−	1	−	1	−	−
NI-siderophore	−	2	2	2	3	−
melanin	−	1	−	1	2	−
NRP-metallophore, NRPS	−	1	−	1	−	1
lanthipeptide-class-iii	−	1	−	1	−	−
T3PKS	−	1	1	3	1	−
Others	7	4	4	9	4	2
**Overall**	**19**	**21**	**17**	**28**	**17**	**16**

*A01 and A96 were identified as Nocardiopsis sp., while A03, A45, A57, and A90 were identified as Streptomyces sp.

*Streptomyces* sp. A03 displayed BGCs encompassing 5 terpene clusters, 2 NRPS clusters, and 2 NI-siderophore clusters and other metabolites. The most prevalent terpene cluster encoded for the production of albaflavenone (similarity 100 %), ebelactone (similarity 5 %), hopene (similarity 100 %), versipelostatin (similarity 5 %) and geosmin (similarity 100 %). *Streptomyces* sp. A45 contained 4 terpene clusters, 3 lassopeptide clusters, 2 NI-siderophore clusters, and other metabolites. The terpene clusters in this isolate were associated with geosmin (similarity 100 %), hopene (similarity 92 %), carotenoid (similarity 54 %), and albaflavenone (similarity 100 %). *Streptomyces* sp. A57 possessed 4 terpene clusters, 3 T3PKS clusters, 2 lanthipeptide-class-i clusters, 2 ectoine clusters, 2 NI- siderphore clusters and other metabolites. The terpene gene clusters in A57 were linked with the production of albaflavenone (similarity 100 %), geosmin (similarity 100 %), hopene (similarity 61 %), and carotenoid (similarity 63 %). *Streptomyces* sp. A90 exhibited 4 terpene clusters, 3 NI-siderophore clusters, 2 T2PKS clusters, 2 melanin clusters, and other metabolites. The terpene gene clusters displayed similarity to hopene (92 %), albaflavenone (100 %), and isorenieratene (100 %).

**Fig. 6:**
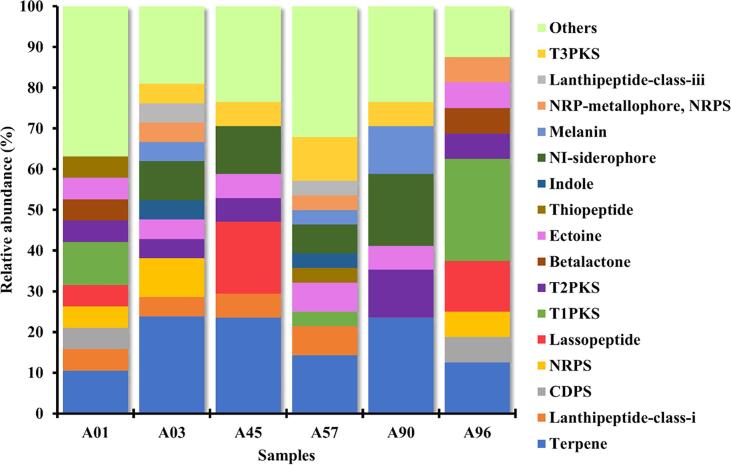
The distribution of BGC types across all *Streptomyces* sp. and *Nocardiopsis* sp. isolates, as predicted by antiSMASH.

*Nocardiopsis* sp. A01 displayed 2 terpene clusters, and 2 T1PKS clusters. The terpene gene clusters were associated with legonindolizidine A6 (similarity 12 %) and isorenieratene (similarity 100 %). *Nocardiopsis* sp. A96 harbored 4 T1PKS clusters, 2 terpene clusters, 2 lassopeptide clusters, and other metabolites. The terpene gene clusters were linked with legonindolizidine A6 (similarity 12 %) and isorenieratene (similarity 100 %) (Fig. 6, Table 3). A list of all the BGCs and their profiles is provided in **Table S4a-f**.

**Table 3:** Clusters and secondary metabolites with 100% similarity to known clusters identified in the genomes of *Nocardiopsis* sp. and *Streptomyces* sp.

**Secondary metabolite**	**Type**	**From**	**To**
***Nocardiopsis* sp. A01**			
LP2006	lassopeptide	18,01,950	18,24,485
isorenieratene	terpene	26,37,004	26,62,551
***Streptomyces* sp. A03**			
albaflavenone	terpene	4,25,113	4,45,709
isorenieratene	NAPAA, terpene	14,35,333	14,87,890
5-dimethylallylindole-3-acetonitrile	indole	16,10,717	16,31,844
SapB	lanthipeptide-class-iii	45,04,206	45,26,815
hopene	terpene	45,92,647	46,18,417
ectoine	ectoine	64,44,517	64,54,915
geosmin	terpene	73,72,825	73,95,023
***Streptomyces* sp. A45**			
geosmin	terpene	2,73,683	2,95,429
citrulassin D	lassopeptide	5,25,969	5,47,865
antimycin	NRPS, T1PKS	16,11,958	16,63,626
ectoine	ectoine	31,83,336	31,92,127
alkylresorcinol	T3PKS	41,72,393	42,13,538
aborycin	lassopeptide	51,72,948	51,92,536
albaflavenone	terpene	53,64,309	53,85,322
streptamidine	LAP	66,54,665	66,77,182
***Streptomyces* sp. A57**			
SapB	lanthipeptide-class-iii	16,98,010	17,20,607
albaflavenone	terpene	25,14,059	25,33,571
ectoine	ectoine	33,08,418	33,17,658
streptamidine	thiopeptide	45,37,559	45,77,941

geosmin	terpene	57,34,333	57,55,238
germicidin	T3PKS, RiPP-like	60,09,074	60,48,423
flaviolin/1,3,6,8-tetrahydroxynaphthalene	T3PKS	61,86,678	62,26,605
scabichelin	NRP-metallophore, NRPS	63,03,707	63,68,311
***Streptomyces* sp. A90**			
γ-butyrolactone	terpene, butyrolactone	13,94,046	14,14,291
ectoine	Ectoine	34,17,648	34,28,046
albaflavenone	Terpene	54,68,874	54,89,193
isorenieratene	Terpene	70,73,596	70,99,159
***Nocardiopsis* sp. A96**			
fuscachelin A/ fuscachelin B/ fuscachelin C	NRP-metallophore, NRPS	4,52,429	5,16,722
LP2006	lassopeptide	30,91,503	31,12,836
isorenieratene	terpene	47,47,856	47,73,403
branched-chain fatty acids	lassopeptide	53,52,297	53,74,566

### 3.5 Determination of sequence types

The *in-silico* MLST analysis of *Streptomyces* sp. samples identified novel allelic profiles, suggesting potential genomic novelty. This analysis identified variations in the sequences of housekeeping genes compared to the known alleles, potentially indicating the presence of novel alleles within the genomes.

*Streptomyces* sp. A03 displayed an allelic profile of 16S_124, atpD_185, recA_156, rpoB_175, and trpB_190. *Streptomyces* sp. A45 exhibited a distinct profile of 16S_88, atpD_91, gyrB_46, recA_185, rpoB_170, and trpB_201. Similarly, *Streptomyces* sp. A57 possessed a unique allelic profile of 16S_100, atpD_165, recA_39, and trpB_148. *Streptomyces* sp. A90 had an allelic profile of 16S_111, atpD_170, recA_99, rpoB_101 and trpB_199 **(**Table 4**)**. These findings indicate the presence of novel allelic profiles in all analyzed *Streptomyces* sp. samples, highlighting the need for further experimental validation.

**Table 4:** MLST profiling of housekeeping genes in *Streptomyces* sp. Samples.

**Locus**	**Identity**	**Coverage**	**Alignment Length**	**Allele Length**	**Gaps**	**Allele**
***Streptomyces* sp. A03**						
16S	99.17788	99.77578	1338	1338	3	16S_124
atpD	99.19192	100	495	495	0	**atpD_185**
recA	97.22222	100	504	504	0	**recA_156**
rpoB	98.33333	100	540	540	0	**rpoB_175**
trpB	95.7672	100	567	567	0	**trpB_190**
***Streptomyces* sp. A45**						
16S	99.53107	63.8951	853	1335	0	16S_88
atpD	99.39394	100	495	495	0	**atpD_91**
gyrB	98.02469	100	405	405	0	**gyrB_46**
recA	98.01587	100	504	504	0	**recA_185**
rpoB	99.44444	100	540	540	0	**rpoB_170**
trpB	96.11993	100	567	567	0	**trpB_201**
***Streptomyces* sp. A57**						
16S	98.27973	99.8502	1337	1335	4	16S_100
atpD	97.77778	100.0	495	495	0	**atpD_165**
recA	96.03175	100.0	504	504	0	**recA_39**
trpB	95.06173	100.0	567	567	0	**trpB_148**
***Streptomyces* sp. A90**						
16S	99.25094	100.0	1335	1335	0	**16S_111**
atpD	98.78788	100.0	495	495	0	**atpD_170**
recA	97.22222	100.0	504	504	0	**recA_99**
rpoB	99.62963	100.0	540	540	0	**rpoB_101**
trpB	95.19231	60.5825	312	515	0	trpB_199

*Bold denotes the novel alleles of genomes ST.

## 4 Discussion

The comprehensive investigation of the genomic potential of *Actinomycetes* isolated from marine sponges and sediments represents a significant contribution to understanding the microbial ecology and biotechnological applications of these organisms. The utilization of Illumina sequencing technology coupled with stringent quality control measures ensures the reliability and accuracy of the obtained sequences.^36^

In our study, all the strains of the *Streptomyces* sp. (A03, A45, A57, and A90) showed a high number of genes classified for transcription and signal transduction mechanism, which aligns with the previous studies on investigation of genes associated with marine adaptations in *Streptomyces* from sponge samples^32^. A comparative study of genomes from marine and non-marine sources found that genes related to post-translational modification, chaperones, and protein turnover (COG O), as well as those involved in translation, ribosomal structure, and biogenesis (COG J), were more abundant in marine-derived genomes than in those from other sources.^37^

The genome mining of secondary metabolites using antiSMASH analysis uncovered diverse BGCs associated with the production of bioactive compounds.^38^ The identification of numerous BGCs, including terpenes, non-ribosomal peptides, polyketides, and siderophores, suggests the metabolic versatility and biotechnological potential of the *Actinomycetes* strains. Additionally, the presence of unique gene clusters highlights the strain-specific metabolic pathways. Legonindolizidine A6, fuscachelin A/ fuscachelin B/ fuscachelin C, purincyclamide, incednine, and LP2006 were uniquely found in *Nocardiopsis* sp. (A01 and A96) and were absent in the genomes of *Streptomyces* sp. (A03, A45, A57, and A90). Conversely, albaflavenone, desferrioxamin B/ desferrioxamine E, and hopene were exclusive to all four *Streptomyces* sp. (A03, A45, A57, and A90) and not found in the *Nocardiopsis* strains. Ectoine was present in all the genomes of both *Nocardiopsis* sp. (A01 and A96) and *Streptomyces* sp. (A03, A45, A57, and A90), which has been previously reported to play a role in adaptation to extreme saline marine environment.^39^ A transcriptomic study on *Nocardiopsis dassonvillei* NCIM 5124 further revealed the upregulation of genes associated with ectoine biosynthesis under salt stress.^40^ Notably, *Streptomyces* sp. A57 was found to produce a β-lactamase inhibitor with 54 % similarity to clauvulanic acid.^41^ Additionally, it harbored a BGC with 52 % similarity to showdomycin, a compound known to inhibit RNA polymerases and demonstrate antimicrobial activity against *Escherichia coli*.^42^, ^43^ Isorenieratene was predicted in the genomes of *Nocardiopsis* sp. (A01, and A96) and *Streptomyces* sp. (A03 and A90) with 100 % similarity. Similarly, a study on Actinobacteria, including the genera *Nocardia*, *Micromonospora*, *Saccharomonospora* and *Streptomyces*, revealed the presence of isorenieratene across all studied species. This compound has been reported to exhibit antiviral, antibacterial and antifungal infections.^44^ Notably, all the *Streptomyces* strains analyzed (A03, A45, A57, and A90) harbored a BGC with 83 % similarity to desferrioxamine E, a siderophore previously predicted and experimentally extracted through high-performance liquid chromatography in a study by Da-Eun et al., along with other secondary metabolites such as spoxazomicins, 6-prenyltryptophol, and N-acetyltryptamine. These findings further support the potential bioactivity of the identified BGC.^45^ Our results add value to the previous experimental studies and also address the mechanism of action which was lacking in the previous study.

Previous study on *Streptomyces* sp. RKND004 has enabled the isolation of bioactive polyether ionophores, namely Terrosamycins A and B from Prince Edward Island sediment, which exhibited significant activity against gram-positive pathogens and against breast cancer cell lines.^46^ The marine-derived piperazimycin from *Streptomyces* sp. (strain CNQ-593) shows excellent activity against tumor cells^47^. Another study reported that chromomycins exhibited strong cytotoxicity and antitumor application against colon cancer cell lines.^48^

Phylogenetic analysis based on 16S rRNA and housekeeping genes elucidated the evolutionary relationships among the *Actinomycetes* isolates and reference strains. Both the 16S rRNA and housekeeping gene phylogenies exhibited a consistent clustering pattern, wherein *Nocardiopsis* sp. A01 clustered with *Nocardiopsis alba*, indicating a close evolutionary relationship. Similarly, *Streptomyces* sp. A03 consistently clustered with *Streptomyces parvulus* in both phylogenetic trees (ANI: 99.25 %), further supporting their relatedness. However, *Streptomyces* sp. A57 exhibited inconsistent clustering across the two phylogenetic trees. Notably, the ANI value between *Streptomyces* sp. A57 and its closest identified species (*Streptomyces broussonetiae*) identified through BLAST was 82.2 %, which is below the established species delineation threshold of 95 %.^49^ This suggests that *Streptomyces* sp. A57 may represent a novel species. A similar pattern of inconsistencies in phylogenetic trees was observed for *Streptomyces* sp. A45 and A90, with their ANI values to the closest species was also less than 95 %. Notably, the MLST analysis provided insights into the genetic diversity and novel sequence types present within the *Streptomyces* sp. isolates, underscoring their evolutionary significance.

Our study provides a comprehensive understanding of the genomic and metabolic potential of *Actinomycetes* isolated from marine sponges and sediments, shedding light on their ecological roles and biotechnological applications. Genome mining of the *Actinomycete* isolates revealed numerous secondary metabolites associated with antimicrobial activities. We found that most of the predicted BGCs from both *Streptomyces* sp. and *Nocardiopsis* sp. showed low similarity to known BGCs, suggesting the potential of these isolates to produce novel secondary metabolites that could be explored for the development of new antibiotics.

## 5 Conclusion

This study provides a comprehensive genomic and functional characterization of *Actinomycetes* isolated from marine sponge samples. High-quality assembled genomes enabled in-depth exploration of their novelty. Notably, *Streptomyces* sp. A57 possessed the highest number of BGCs among all isolates, and antiSMASH analysis revealed low similarity to known clusters, suggesting a rich source of novel secondary metabolites. These findings not only enhance our understanding of *Actinomycete* ecology in marine environments but also lay the groundwork for future exploration of their potential applications in drug discovery and natural product synthesis.

## CRediT authorship contribution statement

**Hithesh Kumar:** Writing – original draft, Methodology, Formal analysis, Data curation. **Santhiya Vijayakumar:** Writing – original draft, Methodology, Formal analysis, Data curation. **Neha Shintre:** Methodology, Formal analysis. **Vaijayanti Tamhane:** Validation, Supervision. **Neelima Deshpande:** Validation, Supervision. **Tushar Joshi:** Validation, Formal analysis. **Shalini Mathpal:** Validation, Formal analysis. **Anand Anbarasu:** Writing – review & editing, Validation, Supervision, Investigation, Funding acquisition. **Sudha Ramaiah:** Writing – review & editing, Validation, Project administration, Funding acquisition, Conceptualization.

## Funding

The authors gratefully acknowledge the Indian Council of Medical Research (10.13039/501100001411ICMR), New Delhi, Government of India agency, for the research grant (IRIS ID: 2021-11889; AMR/Adhoc/290/2022-ECD-II).

## Data Availability

The raw genome sequencing data and genome assembly generated from this study can be found under NCBI Sequence Read Archive (SRA) under the Bio-project ID: **PRJNA1042969.**

## Declaration of competing interest

The authors declare that they have no known competing financial interests or personal relationships that could have appeared to influence the work reported in this paper.
