# Supplementary material for: In silico exploration of biosynthetic gene clusters in marine Streptomyces sp. and Nocardiopsis sp. from the western coast of India: Genome-based profiling using whole genome sequencing
Source: J Genet Eng Biotechnol. 2025 Mar 25;23(2):100483. doi: 10.1016/j.jgeb.2025.100483 (PMC11985029; doi:10.1016/j.jgeb.2025.100483)
Supplement: Supplementary Data 1 [file mmc1.docx]

**Supplementary appendix**

***In silico* exploration of Biosynthetic Gene Clusters in Marine *Streptomyces* sp. and *Nocardiopsis* sp. from the western coast of India: Genome-based Profiling using Whole Genome Sequencing**

**Contents**

**Table S1a:** Raw data Statistics of *Actinomycetes* samples

**Table S1b:** Pre-processed Data Statistics of *Actinomycetes* samples

**Table S1c:** Genome Assembly and Gene Prediction Statistics

**Table S2:** Functional annotation of the predicted gene product of *Nocardiopsis* sp. *Streptomyces* sp.

**Table S3:** NCBI BLAST analysis results of 16S rRNA sequences against NR database

**Table S4a:** BGC profile of *Nocardiopsis* sp. A01

**Table S4b:** BGC profile of *Nocardiopsis* sp. A96

**Table S4c:** BGC profile of *Streptomyces* sp. A03

**Table S4d:** BGC profile of *Streptomyces* sp. A45

**Table S4e:** BGC profile of *Streptomyces* sp. A57

**Table S4f:** BGC profile of *Streptomyces* sp. A90

**Figure S1:** (a) Mean quality scores of raw genomic reads for all samples (b) Mean quality scores of pre-processed (trimmed) genomic reads for all samples

**Figure S2:** Genome completeness report of all *Actinomycete* samples using BUSCO

**Figure S3:** Average Nucleotide Identity (ANI) comparison of (a) *Streptomyces* sp. and (b) *Nocardiopsis* sp. with their top corresponding reference genomes identified through BLAST analysis

**Table S1a:** Raw data Statistics of *Actinomycetes* samples

| **Sample name** | **Format** | **Type** | **num_seqs** | **sum_len** | **length of sequence** | **sum_gap** | **Q20(%)** | **Q30(%)** | **GC(%)** |
| --- | --- | --- | --- | --- | --- | --- | --- | --- | --- |
| *Nocardiopsis* sp. A01 | FASTQ | DNA | 4,28,77,822 | 6,43,16,73,300 | 150x2 | 0 | 97.77 | 94.19 | 69 |
| *Streptomyces* sp. A03 | FASTQ | DNA | 3,52,09,704 | 5,31,66,65,304 | 151x2 | 0 | 97.26 | 92.915 | 72 |
| *Streptomyces* sp. A45 | FASTQ | DNA | 4,02,76,180 | 6,04,14,27,000 | 150x2 | 0 | 97.5 | 93.55 | 72 |
| *Streptomyces* sp. A57 | FASTQ | DNA | 3,89,44,812 | 5,84,17,21,800 | 150x2 | 0 | 95.685 | 90.05 | 71 |
| *Streptomyces* sp. A90 | FASTQ | DNA | 3,43,52,100 | 5,18,71,67,100 | 151x2 | 0 | 97.105 | 92.535 | 71 |
| *Nocardiopsis* sp. A96 | FASTQ | DNA | 4,15,62,650 | 6,23,43,97,500 | 150x2 | 0 | 97.57 | 93.8 | 69 |

**Table S1b:** Pre-processed Data Statistics of *Actinomycetes* samples

| **Sample name** | **Format** | **Type** | **num_seqs** | **sum_len** | **length of sequence** | **sum_gap** | **Q20(%)** | **Q30(%)** | **GC (%)** |
| --- | --- | --- | --- | --- | --- | --- | --- | --- | --- |
| *Nocardiopsis* sp. A01 | FASTQ | DNA | 4,23,62,838 | 6,28,02,00,003 | 148.25x2 | 0 | 98.495 | 95.2 | 69 |
| *Streptomyces* sp. A03 | FASTQ | DNA | 3,38,51,006 | 5,04,76,78,810 | 149.15x2 | 0 | 98.205 | 94.26 | 72 |
| *Streptomyces* sp. A45 | FASTQ | DNA | 3,97,12,698 | 5,87,81,07,353 | 148.05x2 | 0 | 98.33 | 94.705 | 71 |
| *Streptomyces* sp. A57 | FASTQ | DNA | 3,69,99,676 | 5,29,56,12,113 | 143.1x2 | 0 | 98.07 | 93.565 | 71 |
| *Streptomyces* sp. A90 | FASTQ | DNA | 3,29,75,794 | 4,91,65,25,573 | 149.1x2 | 0 | 98.085 | 93.92 | 71 |
| *Nocardiopsis* sp. A96 | FASTQ | DNA | 4,09,75,404 | 6,06,95,98,415 | 148.15x2 | 0 | 98.375 | 94.92 | 69 |

**Table S1c:** Genome Assembly and Gene Prediction Statistics

| **Sample name** | **Scaffolds Generated** | **Total Scaffolds Length** | **Total Number of Non-ATGC Characters** | **N50 Value** | **GC Content** | **Gene/mRNA** | **mRNA** | **CDS** | **CRISPR** | **misc_RNA** | **rRNA** | **tRNA** | **tmRNA** |
| --- | --- | --- | --- | --- | --- | --- | --- | --- | --- | --- | --- | --- | --- |
| *Nocardiopsis* sp. A01 | 75 | 5860446 | 410 | 692549 | 69.7 | 5201 | 5201 | 5096 | - | 26 | 5 | 73 | 1 |
| *Streptomyces* sp. A03 | 108 | 7780734 | 230 | 326105 | 72.69 | 7018 | 7018 | 6859 | - | 74 | 7 | 77 | 1 |
| *Streptomyces* sp. A45 | 64 | 7468284 | 110 | 471507 | 72.64 | 6706 | 6706 | 6569 | - | 45 | 4 | 87 | 1 |
| *Streptomyces* sp. A57 | 260 | 7858046 | 1300 | 184991 | 79.48718 | 7245 | 7245 | 7111 | - | 49 | 4 | 80 | 1 |
| *Streptomyces* sp. A90 | 97 | 7595990 | 210 | 267143 | 52.5641 | 6997 | 6997 | 6862 | - | 49 | 6 | 79 | 1 |
| *Nocardiopsis* sp. A96 | 28 | 6032168 | 400 | 617594 | 69.73 | 5390 | 5390 | 5289 | - | 27 | 3 | 70 | 1 |

**Table S2:** Functional annotation of the predicted gene product of *Nocardiopsis* sp. *Streptomyces* sp.

| **LETTER** | **DESCRIPTION** | **A01** | **A03** | **A45** | **A57** | **A90** | **A96** |
| --- | --- | --- | --- | --- | --- | --- | --- |
| A | RNA processing and modification | 1 | 1 | 1 | 1 | 1 | 1 |
| B | Chromatin structure and dynamics | 0 | 0 | 0 | 0 | 0 | 0 |
| C | Energy production and conversion | 221 | 301 | 313 | 311 | 326 | 221 |
| D | Cell cycle control, cell division, chromosome partitioning | 68 | 87 | 94 | 83 | 86 | 73 |
| E | Amino acid transport and metabolism | 352 | 445 | 401 | 419 | 428 | 354 |
| F | Nucleotide transport and metabolism | 121 | 128 | 113 | 126 | 116 | 125 |
| G | Carbohydrate transport and metabolism | 304 | 551 | 506 | 542 | 565 | 301 |
| H | Coenzyme transport and metabolism | 273 | 346 | 326 | 299 | 336 | 282 |
| I | Lipid transport and metabolism | 222 | 307 | 297 | 330 | 299 | 245 |
| J | Translation, ribosomal structure and biogenesis | 245 | 272 | 269 | 271 | 266 | 252 |
| K | Transcription | 435 | 589 | 544 | 595 | 624 | 439 |
| L | Replication, recombination and repair | 128 | 160 | 144 | 169 | 160 | 123 |
| M | Cell wall/membrane/envelope biogenesis | 182 | 287 | 265 | 291 | 270 | 189 |
| N | Cell motility | 13 | 16 | 11 | 14 | 13 | 11 |
| O | Posttranslational modification, protein turnover, chaperones | 156 | 193 | 199 | 204 | 220 | 162 |
| P | Inorganic ion transport and metabolism | 195 | 230 | 211 | 240 | 218 | 200 |
| Q | Secondary metabolites biosynthesis, transport and catabolism | 151 | 215 | 200 | 192 | 155 | 165 |
| R | General function prediction only | 323 | 426 | 411 | 414 | 423 | 330 |
| S | Function unknown | 155 | 201 | 187 | 205 | 212 | 161 |
| T | Signal transduction mechanisms | 269 | 463 | 432 | 423 | 445 | 281 |
| U | Intracellular trafficking, secretion, and vesicular transport | 36 | 54 | 48 | 52 | 54 | 37 |
| V | Defense mechanisms | 152 | 167 | 180 | 161 | 144 | 154 |
| W | Extracellular structures | 2 | 2 | 3 | 2 | 3 | 2 |
| X | Mobilome: prophages, transposons | 17 | 23 | 32 | 64 | 15 | 29 |
| Y | Nuclear structure | 0 | 0 | 0 | 0 | 0 | 0 |
| Z | Cytoskeleton | 0 | 1 | 0 | 0 | 0 | 0 |

*A01, and A96 belongs to Nocardiopis sp. and A03, A45, A57, and A90 belongs to Streptomyces sp.*

**Table S3:** NCBI BLAST analysis results of 16S rRNA sequences against NR database

| **S.No** | **Description** | **Scientific Name** | **Accession** |
| --- | --- | --- | --- |
| **Top BLAST Hits of 16S rRNA Sequences from *Streptomyces* sp. against the NCBI NR Database** | | | |
| 1 | *Streptomyces parvulus* strain 2297, complete genome | *Streptomyces parvulus* | CP015866.1 |
| 2 | *Streptomyces olivaceus* strain KLBMP 5084 chromosome, complete genome | *Streptomyces olivaceus* | CP016795.1 |
| 3 | *Streptomyces nigra* strain LM01 chromosome | *Streptomyces nigra* | CP147865.1 |
| 4 | *Streptomyces pactum* strain ACT12, complete genome | *Streptomyces pactum* | CP019724.1 |
| 5 | *Streptomyces iakyrus* strain CGMCC 4.1912 chromosome, complete genome | *Streptomyces iakyrus* | CP143088.1 |
| 6 | *Streptomyces actuosus* strain ATCC 25421 chromosome, complete genome | *Streptomyces actuosus* | CP029788.1 |
| 7 | *Streptomyces violaceus* strain NBC_00293 chromosome, complete genome | *Streptomyces violaceus* | CP108342.1 |
| 8 | *Streptomyces purpurascens* strain NBC_00017 chromosome, complete genome | *Streptomyces purpurascens* | CP108341.1 |
| 9 | *Streptomyces coelicoflavus* strain NBC_00357 chromosome, complete genome | *Streptomyces coelicoflavus* | CP107977.1 |
| 10 | *Streptomyces janthinus* strain JCM 4387 chromosome, complete genome | *Streptomyces janthinus* | CP134213.1 |
| 11 | *Streptomyces rubrogriseus* strain NBRC 15455 chromosome, complete genome | *Streptomyces rubrogriseus* | CP116256.1 |
| 12 | *Streptomyces lienomycini* strain DSM 41475 chromosome, complete genome | *Streptomyces lienomycini* | CP116257.1 |
| 13 | *Streptomyces coelicolor* strain M1154/pAMX4/pGP1416 chromosome, complete genome | *Streptomyces coelicolor* | CP050522.1 |
| 14 | *Streptomyces anthocyanicus* strain NBC 01687 chromosome, complete genome | *Streptomyces anthocyanicus* | CP109205.1 |
| 15 | *Streptomyces tuirus* JCM 4255 DNA, complete sequence | *Streptomyces tuirus* | AP023439.1 |
| 16 | *Streptomyces coeruleorubidus* strain ATCC 13740 chromosome, complete genome | *Streptomyces coeruleorubidus* | CP023694.1 |
| 17 | *Streptomyces alboflavus* strain MDJK44 chromosome, complete genome | *Streptomyces alboflavus* | CP021748.1 |
| 18 | *Streptomyces flavofungini* strain TRM90047 chromosome, complete genome | *Streptomyces flavofungini* | CP128846.1 |
| 19 | *Streptomyces cellulosae* strain NBC_01607 chromosome, complete genome | *Streptomyces cellulosae* | CP109308.1 |
| 20 | *Streptomyces thermocarboxydus* strain K155 chromosome | *Streptomyces thermocarboxydus* | CP058273.1 |
| 21 | *Streptomyces spectabilis* strain ATCC 27465 chromosome, complete genome | *Streptomyces spectabilis* | CP023690.1 |
| 22 | *Streptomyces leeuwenhoekii* strain C34 (= DSM 42122 = NRRL B-24963) genome assembly, chromosome: chromosome | *Streptomyces leeuwenhoekii* | LN831790.1 |

***Table S1 continued***

| 23 | *Streptomyces huasconensis* strain D23 chromosome, complete genome | *Streptomyces huasconensis* | CP086119.1 |
| --- | --- | --- | --- |
| 24 | *Streptomyces cyaneogriseus* subsp. noncyanogenus strain NMWT 1, complete genome | *Streptomyces cyaneogriseus subsp. noncyanogenus* | CP010849.1 |
| 25 | *Streptomyces luteogriseus* strain NBC_00712 chromosome, complete genome | *Streptomyces luteogriseus* | CP108993.1 |
| 26 | *Streptomyces broussonetiae* strain T44 chromosome | *Streptomyces broussonetiae* | CP047020.1 |
| 27 | *Streptomyces collinus* strain L2 chromosome, complete genome | *Streptomyces collinus* | CP052033.1 |
| 28 | *Streptomyces pseudovenezuelae* strain NBC_00598 chromosome, complete genome | *Streptomyces pseudovenezuelae* | CP107755.1 |
| 29 | *Streptomyces venezuelae* strain ATCC 14585 chromosome, complete genome | *Streptomyces venezuelae* | CP029191.1 |
| 30 | *Streptomyces phaeoluteigriseus* strain Qhu-M197 chromosome, complete genome | *Streptomyces phaeoluteigriseus* | CP099468.1 |
| 31 | *Streptomyces spinosirectus* strain CRSS-Y-16 chromosome, complete genome | *Streptomyces spinosirectus* | CP090447.1 |
| 32 | *Streptomyces hawaiiensis* strain ATCC 12236 chromosome, complete genome | *Streptomyces hawaiiensis* | CP021978.1 |
| 33 | *Streptomyces aquilus* strain GGCR-6 chromosome, complete genome | *Streptomyces aquilus* | CP034463.1 |
| 34 | *Streptomyces fodineus* strain TW1S1 chromosome, complete genome | *Streptomyces fodineus* | CP017248.1 |
| 35 | *Streptomyces canus* strain NBC 00349 chromosome, complete genome | *Streptomyces canus* | CP107989.1 |
| 36 | *Streptomyces chartreusis* strain NBC_00215 chromosome, complete genome | *Streptomyces chartreusis* | CP108114.1 |
| 37 | *Streptomyces bobili* strain NBC_00302 chromosome, complete genome | *Streptomyces bobili* | CP108038.1 |
| 38 | *Streptomyces berlinensis* strain 14.2 chromosome | *Streptomyces berlinensis* | CP115393.1 |
| 39 | *Streptomyces fungicidicus* strain TXX3120 chromosome, complete genome | *Streptomyces fungicidicus* | CP023407.1 |
| 40 | *Streptomyces althioticus* strain NBC 01666 chromosome, complete genome | *Streptomyces althioticus* | CP109234.1 |
| 41 | *Streptomyces lusitanus* strain FZ202 chromosome | *Streptomyces lusitanus* | CP104864.1 |
| 42 | *Streptomyces glaucescens* strain GLA.O, complete genome | *Streptomyces glaucescens* | CP009438.1 |
| 43 | *Streptomyces ambofaciens* strain DSM 40697 chromosome, complete genome | *Streptomyces ambofaciens* | CP012949.1 |
| 44 | *Streptomyces chromofuscus* strain DSM 40273 chromosome, complete genome | *Streptomyces chromofuscus* | CP063374.1 |
| **Top BLAST Hits of 16S rRNA Sequences from *Nocardiopsis* sp. against the NCBI NR Database** | | | |
| 45 | *Nocardiopsis alba* ATCC BAA-2165, complete genome | *Nocardiopsis alba* ATCC BAA-2165 | CP003788.1 |
| 46 | *Nocardiopsis exhalans* strain JCM11759T chromosome, complete genome | *Nocardiopsis exhalans* | CP099837.1 |
| 47 | Nocardiopsis changdeensis strain Mg02 chromosome, complete genome | Nocardiopsis changdeensis | CP074133.1 |
| 48 | Nocardiopsis dassonvillei strain HZNU_N_1, complete genome | Nocardiopsis dassonvillei | CP022434.1 |

**Table S3a:** BGC profile of *Nocardiopsis* sp. A01

| **Type** | **Identified BGC** | **Compound class** | **Similarity** | **From** | **To** |
| --- | --- | --- | --- | --- | --- |
| NRPS, NRPS-like | acyldepsipeptide 1 | NRP+Polyketide | 15% | 3 | 31,721 |
| terpene | legonindolizidine A6 | NRP+Alkaloid | 12% | 1,21,118 | 1,37,929 |
| NRP-mettalophore, NRPS, ectoine | fuscachelin A/ fuscachelin B/ fuscachelin C | NRP | 88% | 3,23,948 | 3,89,055 |
| lanthipeptide-class-i | arsono-polyketide | Polyketide | 12% | 4,38,152 | 4,62,211 |
| CDPS | purincyclamide | Other | 40% | 5,23,623 | 5,44,309 |
| NRPS | incednine | Polyketide | 4% | 10,20,615 | 10,61,608 |
| lassopeptide | LP2006 | RiPP | 100% | 18,01,950 | 18,24,485 |
| T1PKS | filipin | Polyketide | 46% | 21,64,358 | 22,19,490 |
| terpene | isorenieratene | Terpene | 100% | 26,37,004 | 26,62,551 |
| T2PKS | formicamycins A-M | Polyketide | 9% | 27,02,099 | 27,74,576 |
| betalactone | oxalomycin B | NRP+Polyketide | 6% | 29,66,298 | 29,98,500 |
| thiopeptide, LAP, RRE-containing | nocardiopsistin A/ nocardiopsistin B/ nocardiopsistin C | Polyketide | 13% | 33,12,396 | 33,87,687 |
| ectoine |  |  |  | 36,49,240 | 36,77,528 |
| thiopeptide | ectoine | Other:Ectoine | 75% | 42,93,062 | 43,03,463 |
| T1PKS | TP-1161 | RiPP:Thiopeptide | 79% | 43,97,943 | 44,31,442 |
| thiopeptide, LAP, NRPS | saquayamycin A | Polyketide | 7% | 44,56,214 | 44,99,111 |
| oligosaccharide, lanthipeptide-class-iii | omnipeptin | NRP:Cyclic depsipeptide | 9% | 52,74,145 | 53,40,638 |
| oligosaccharide, NRPS | rubradirin | Polyketide | 6% | 54,26,547 | 54,74,465 |
| other | atratumycin | NRP | 7% | 58,18,274 | 58,60,446 |

**Table S3b:** BGC profile of *Nocardiopsis* sp. A96

| **Type** | **Identified BGC** | **Compound class** | **Similarity** | **From** | **To** |
| --- | --- | --- | --- | --- | --- |
| CDPS | purincyclamide | Other | 40% | 2,76,398 | 2,97,084 |
| NRP-metallophore, NRPS | fuscachelin A/ fuscachelin B/ fuscachelin C | NRP | 100% | 4,52,429 | 5,16,722 |
| terpene | legonindolizidine A6 | NRP+Alkaloid | 12% | 7,28,005 | 7,45,118 |
| NRPS | incednine | Polyketide | 4% | 15,36,971 | 15,81,161 |
| T2PKS, thiopeptide, LAP, RRE-containing | fredericamycin A | Polyketide:Type II polyketide | 45% | 20,66,995 | 21,45,412 |
| T1PKS | saquayamycin A | Polyketide | 7% | 24,30,808 | 24,71,997 |
| ectoine | ectoine | Other:Ectoine | 75% | 26,03,993 | 26,14,394 |
| T1PKS | polyoxypeptin | NRP+Polyketide | 10% | 27,20,940 | 27,76,429 |
| lassopeptide | LP2006 | RiPP | 100% | 30,91,503 | 31,12,836 |
| T1PKS | efomycin K / efomycin L | Polyketide | 45% | 34,89,481 | 35,66,961 |
| betalactone | oxalomycin B | NRP+Polyketide | 6% | 44,05,039 | 44,37,254 |
| T2PKS | formicamycins A-M | Polyketide | 9% | 46,37,416 | 47,09,893 |
| terpene | isorenieratene | Terpene | 100% | 47,47,856 | 47,73,403 |
| T1PKS | kedarcidin | NRP+Polyketide:Iterative type I polyketide+Polyketide: Enediyne type I polyketide | 12% | 50,90,503 | 51,36,319 |
| arylpolyene, thiopeptide, LAP, lassopeptide | enteromycin | Polyketide+NRP | 12% | 52,25,725 | 53,13,612 |
| lassopeptide | branched-chain fatty acids |  | 100% | 53,52,297 | 53,74,566 |

**Table S3c:** BGC profile of *Streptomyces* sp. A03

| **Type** | **Identified BGC** | **Compound class** | **Similarity** | **From** | **To** |
| --- | --- | --- | --- | --- | --- |
| other | cervinomycin B3/ cervinomycin C1/cervinomycin C3/ cervinomycin C4 | Alkaloid | 17% | 21,253 | 61,357 |
| T2PKS | spore pigment | Polyketide | 66% | 3,21,379 | 3,93,554 |
| terpene | albaflavenone | Terpene | 100% | 4,25,113 | 4,45,709 |
| terpene | ebelactone | Polyketide | 5% | 13,86,999 | 14,05,230 |
| NAPAA, terpene | isorenieratene | Terpene | 100% | 14,35,333 | 14,87,890 |
| indole | 5-dimethylallylindole-3-acetonitrile | Other | 100% | 16,10,717 | 16,31,844 |
| NRPS | griseoviridin/fijimycin A | NRP:Cyclic depsipeptide+Polyketide: Trans-AT type I polyketide | 5% | 18,96,578 | 19,45,772 |
| NI-siderophore | grincamycin | Polyketide: Type II polyketide+Saccharide:Hybrid/tailoring saccharide | 5% | 20,04,855 | 20,17,136 |
| lanthipeptide-class-i |  |  |  | 22,36,486 | 22,61,621 |
| melanin | istamycin | Saccharide | 4% | 27,82,399 | 27,92,905 |
| NRP-metallophore, NRPS | coelichelin | NRP | 90% | 37,69,524 | 38,27,874 |
| NI-siderophore | desferrioxamin B/ desferrioxamine E | Other | 83% | 39,63,149 | 39,74,918 |
| NRPS, T1PKS, other, T2PKS, butyrolactone, NRPS-like | polyoxypeptin | NRP+Polyketide | 48% | 42,19,179 | 45,01,338 |
| lanthipeptide-class-iii | SapB | RiPP:Lanthipeptide | 100% | 45,04,206 | 45,26,815 |
| terpene | hopene | Terpene | 100% | 45,92,647 | 46,18,417 |
| terpene | versipelostatin | polyketide | 5% | 60,33,475 | 60,54,328 |
| T3PKS | hexacosalactone | Other | 6% | 60,61,269 | 61,02,453 |

*Table S3c continued*

| ectoine | ectoine | Other | 100% | 64,44,517 | 64,54,915 |
| --- | --- | --- | --- | --- | --- |
| other, NRPS | actinomycin D | NRP | 82% | 65,20,407 | 65,86,399 |
| terpene | geosmin | Terpene | 100% | 73,72,825 | 73,95,023 |
| NRPS | vazabitide A | NRP | 30% | 74,81,968 | 75,57,409 |

**Table S3d:** BGC profile of *Streptomyces* sp. A45

| **Type** | **Identified BGC** | **Compound class** | **Similarity** | **From** | **To** |
| --- | --- | --- | --- | --- | --- |
| terpene | geosmin | Terpene | 100% | 2,73,683 | 2,95,429 |
| NI-siderophore | grincamycin | Polyketide:TYPE II polyketide+Saccharide:Hybrid/tailoring saccharide | 11% | 4,34,066 | 4,46,933 |
| lassopeptide | citrulassin D | RiPP | 100% | 5,25,969 | 5,47,865 |
| terpene | hopene | Terpene | 92% | 9,06,950 | 9,32,795 |
| NRPS | cyclofaulknamycin | Polyketide | 8% | 12,81,306 | 13,57,685 |
| NRPS, T1PKS | antimycin | NRP+Polyketide | 100% | 16,11,958 | 16,63,626 |
| NI-siderophore | desferrioxamin B/ desferrioxamine E | Other | 83% | 22,61,591 | 22,73,363 |
| ectoine | ectoine | Other | 100% | 31,83,336 | 31,92,127 |
| T2PKS | spore pigment | Polyketide | 83% | 38,84,694 | 39,56,488 |
| lanthipeptide-class-i |  |  |  | 41,15,485 | 41,38,771 |
| T3PKS | alkylresorcinol | Polyketide | 100% | 41,72,393 | 42,13,538 |
| terpene | carotenoid | Terpene | 54% | 44,27,925 | 44,49,858 |
| lassopeptide | aborycin | RiPP | 100% | 51,72,948 | 51,92,536 |
| terpene | albaflavenone | Terpene | 100% | 53,64,309 | 53,85,322 |
| lassopeptide | lagmysin | RiPP | 80% | 55,75,984 | 55,98,642 |
| lanthipeptide-class-I, lanthipeptide-class-ii, NRPS, hglE-KS, T1PKS, T2PKS, ectoine, transAT-PKS, PKS-like | kosinostatin | NRP+Polyketide: Type II polyketide+Saccharide:Hybrid/tailoring saccharide | 77% | 56,02,083 | 58,76,762 |
| LAP | streptamidine | RiPP:Other | 100% | 66,54,665 | 66,77,182 |

**Table S3e:** BGC profile of *Streptomyces* sp. A57

| **Type** | **Identified BGC** | **Compound class** | **Similarity** | **From** | **To** |
| --- | --- | --- | --- | --- | --- |
| T3PKS | s56-p1 | NRP | 11% | 1,49,767 | 1,88,641 |
| NI-siderophore |  |  |  | 7,23,804 | 7,35,316 |
| other | asukamycin | Polyketide:Type II polyketide | 3% | 7,55,019 | 7,94,303 |
| lanthipeptide-class-iii | SapB | RiPP:Lanthipeptide | 100% | 16,98,010 | 17,20,607 |
| melanin | istamycin | Saccharide | 5% | 19,60,261 | 19,70,692 |
| NI-siderophore | desferrioxamin B/ desferrioxamine E | Other | 83% | 20,59,041 | 20,70,816 |
| terpene | albaflavenone | Terpene | 100% | 25,14,059 | 25,33,571 |
| blactam | clavulanic acid | Other:Non-NRP beta-lactam | 54% | 26,13,049 | 26,36,614 |
| ectoine | ectoine | Other | 100% | 33,08,418 | 33,17,658 |
| indole | 5-isoprenylindole-3-carboxylate b-D-glycosyl ester | Other | 23% | 38,48,655 | 38,69,782 |
| transAT-PKS | bacillaene | Polyketide+NRP | 14% | 38,80,360 | 39,59,610 |
| T1PKS, NRPS, LAP, thiopeptide | rimosamide | NRP | 35% | 41,85,464 | 42,66,882 |
| ectoine | showdomycin | Other | 52% | 42,71,927 | 42,82,301 |
| other | petrichorin A/ petrichorin B | NRP | 5% | 42,86,648 | 43,26,646 |
| thiopeptide | streptamidine | RiPP:Other | 100% | 45,37,559 | 45,77,941 |
| butyrolactone |  |  |  | 48,29,328 | 48,37,396 |
| T1PKS | 4-hexadecanoyl-3-hydroxy-2-(hydroxymethyl)-2H-furan-5-one | Polyketide | 90% | 51,45,453 | 51,88,701 |
| lanthipeptide-class-i |  |  |  | 52,26,337 | 52,50,760 |
| NRPS-like, NRP-metallophore, NRPS, betalactone, butyrlactone | griseobactin | NRP | 53% | 53,55,309 | 54,16,459 |
| terpene | geosmin | Terpene | 100% | 57,34,333 | 57,55,238 |
| terpene | hopene | Terpene | 61% | 59,49,250 | 59,75,309 |

***Table S3e continued***

| T3PKS | germicidin | Other | 100% | 60,09,074 | 60,48,423 |
| --- | --- | --- | --- | --- | --- |
| T3PKS | flaviolin/1,3,6,8-tetrahydroxynaphthalene | Polyketide | 100% | 61,86,678 | 62,26,605 |
| NRP-metallophore, NRPS | scabichelin | NRP | 100% | 63,03,707 | 63,68,311 |
| other, T3PKS, terpene | merochlorin A/ merochlorinB/ deschloro-merochlorin A/ deschloro-merochlorin B/ isochloro-merochlorin B/ dichloro-merochlorin B/merochlorin D/ merochlorin C | Terpene+Polyketide: Type III polyketide | 78% | 65,19,256 | 65,83,774 |
| terpene | carotenoid | Terpene | 63% | 73,98,694 | 74,24,434 |
| lanthipeptide-class-i | aborycin | Other | 14% | 76,84,625 | 77,09,784 |
| furan, butyrolactone | lactonamycin | Polyketide | 3% | 75,54,753 | 75,77,877 |

**Table S3f:** BGC profile of *Streptomyces* sp. A90

| **Type** | **Identified BGC** | **Compound class** | **Similarity** | **From** | **To** |
| --- | --- | --- | --- | --- | --- |
| melanin | istamycin | Saccharide | 4% | 10,66,974 | 10,77,124 |
| NI-siderophore | desferrioxamin B/ desferrioxamine E | Other | 83% | 11,57,315 | 11,68,061 |
| T2PKS | kinamycin | Polyketide | 36% | 13,03,924 | 13,75,532 |
| terpene, butyrolactone | γ-butyrolactone | Other | 100% | 13,94,046 | 14,14,291 |
| NI-siderophore | grincamycin | Polyketide:TYPE II polyketide+Saccharide:Hybrid/tailoring saccharide | 5% | 15,39,049 | 15,51,154 |
| T2PKS | spore pigment | Polyketide | 75% | 25,74,745 | 26,47,263 |
| ectoine | ectoine | Other | 100% | 34,17,648 | 34,28,046 |
| melanin | melanin | Other | 57% | 36,46,068 | 36,56,442 |
| T1PKS, NRPS | foxicin A/foxicin B/ foxicin C/ foxicin | NRP+Polyketide | 12% | 37,04,299 | 37,82,817 |
| T3PKS | lipopeptide 8D1-1/lipopeptide 8D1-2 | NRP | 11% | 40,85,471 | 41,26,655 |
| terpene |  |  |  | 44,76,226 | 44,95,239 |
| terpene | hopene | Terpene | 92% | 47,73,468 | 47,99,407 |
| NI-siderophore |  |  |  | 54,26,978 | 54,38,798 |
| terpene | albaflavenone | Terpene | 100% | 54,68,874 | 54,89,193 |
| T1PKS,NRPS | arylomycin | NRP:Lipopeptide | 33% | 58,95,759 | 59,66,095 |
| butyrolactone, terpene | maduralactomycin A/ maduralactomycin B/ actinospirol A/ actinospirol B | Polyketide | 22% | 67,97,444 | 68,20,740 |
| terpene | isorenieratene | Terpene | 100% | 70,73,596 | 70,99,159 |


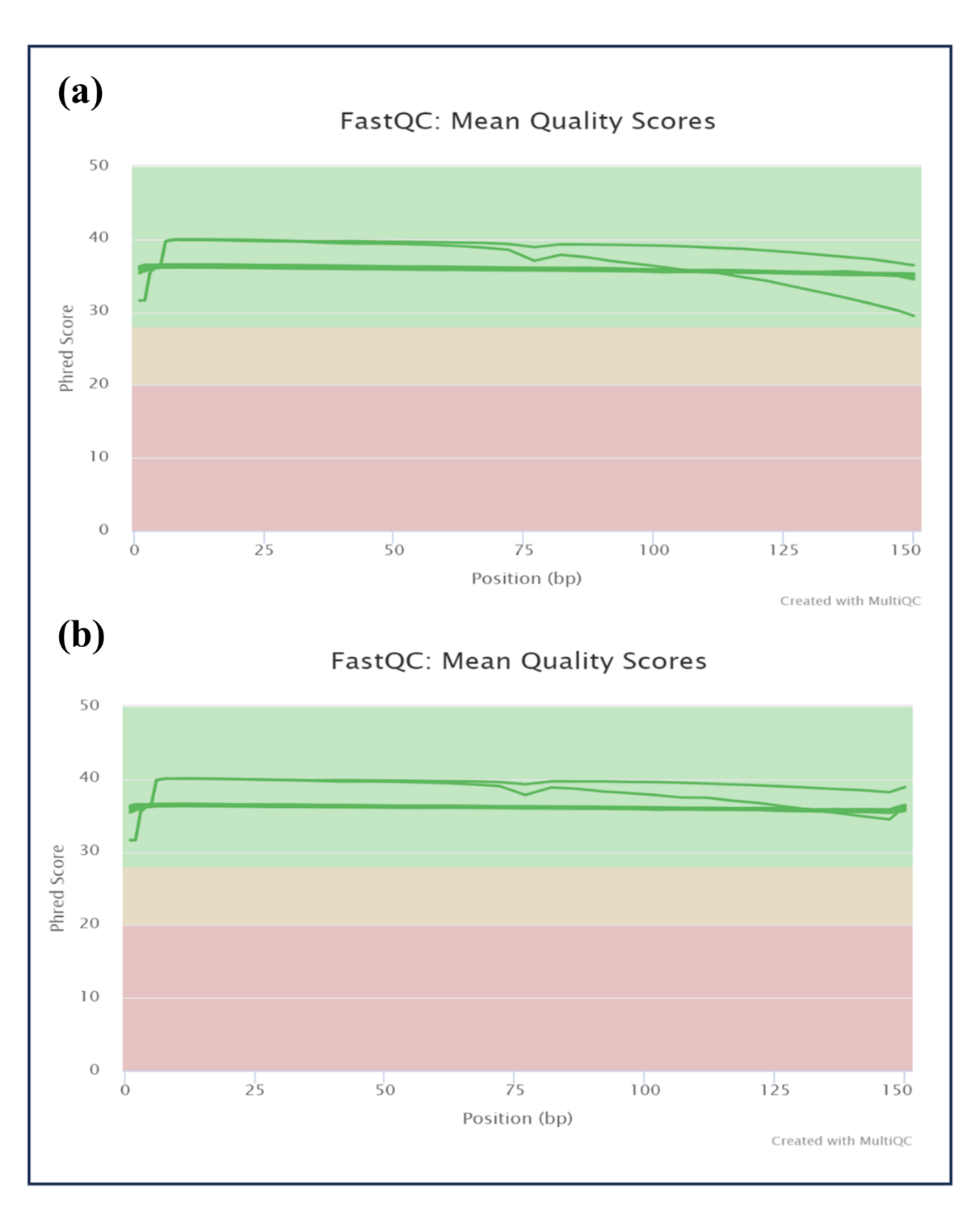


**Figure S1:** (a) Mean quality scores of raw genomic reads for all samples (b) Mean quality scores of pre-processed (trimmed) genomic reads for all samples


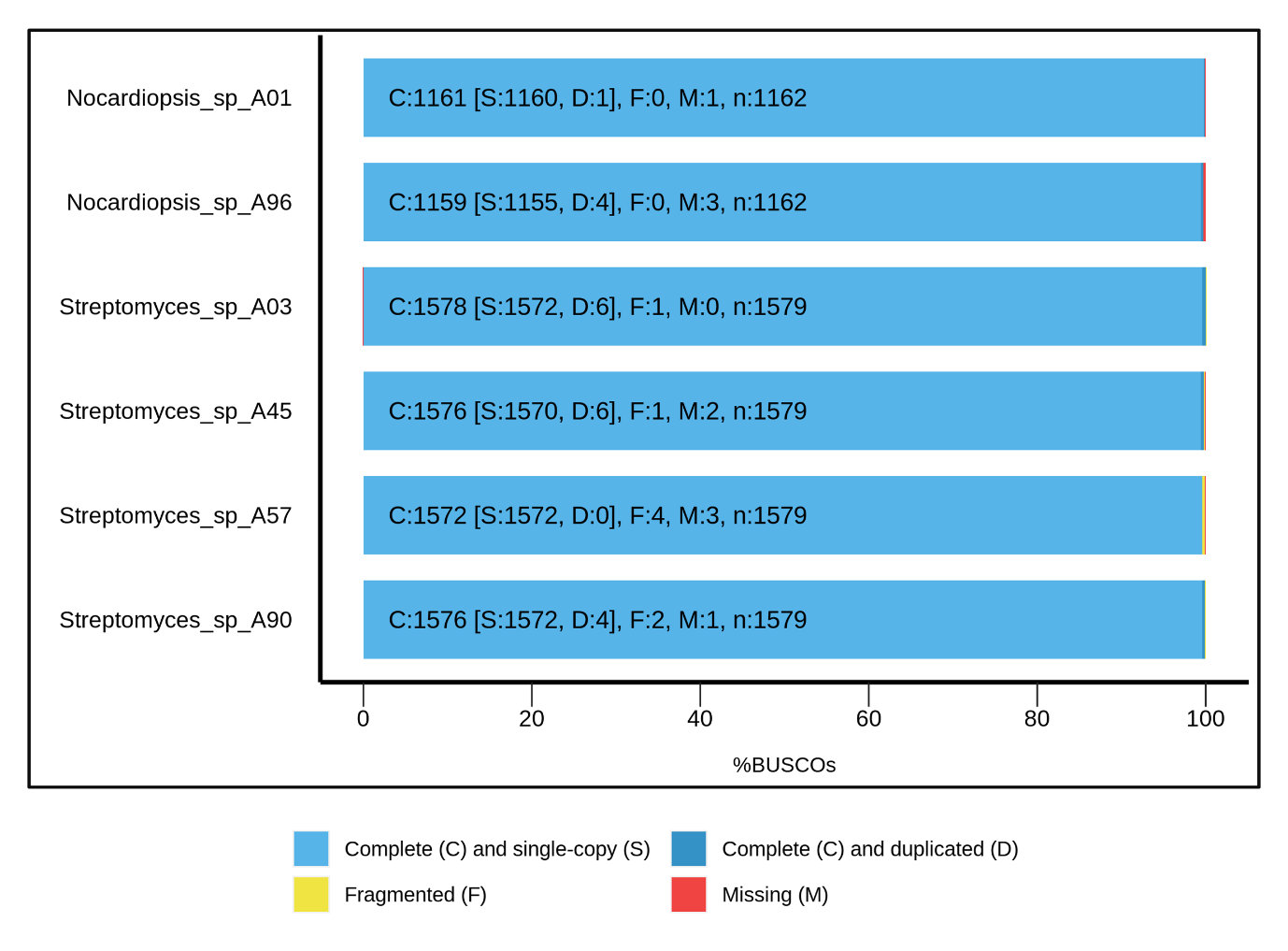


**Figure S2:** Genome completeness report of all *Actinomycete* samples using BUSCO


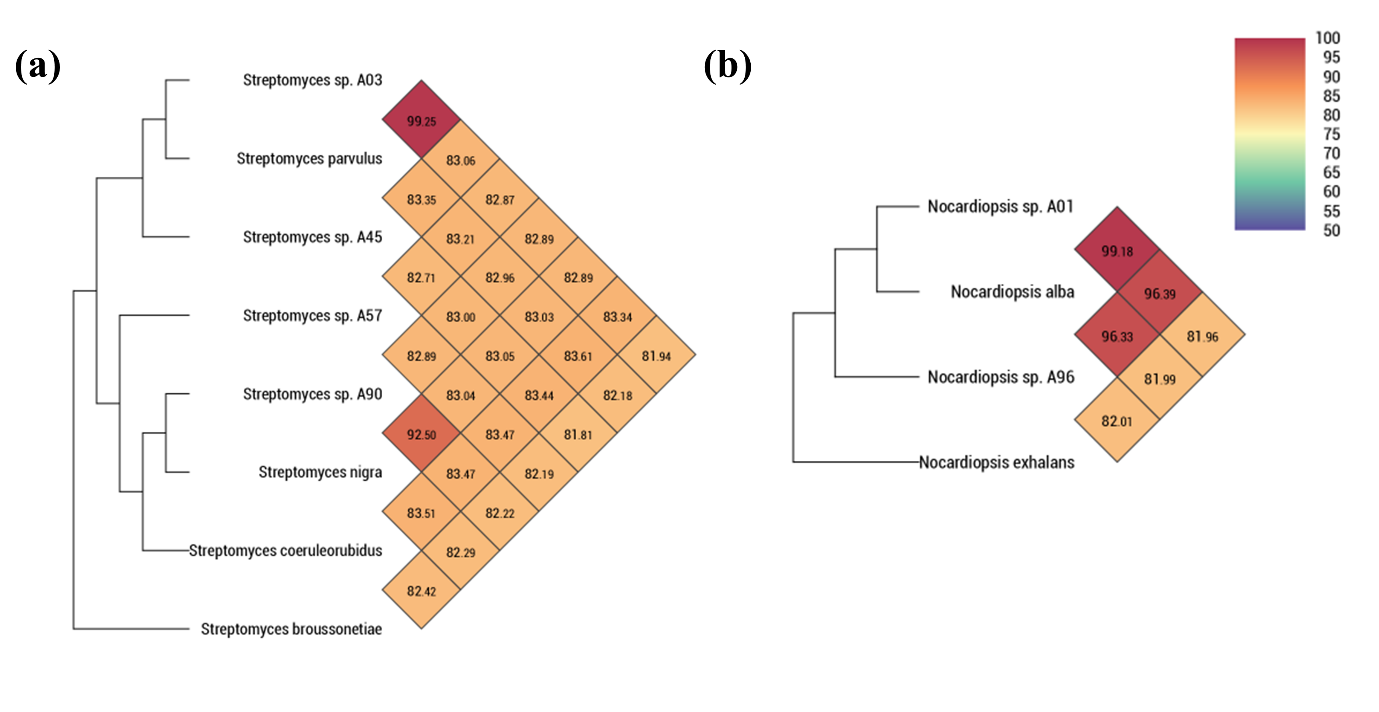


**Figure S3:** Average Nucleotide Identity (ANI) comparison of (a) *Streptomyces* sp. and (b) *Nocardiopsis* sp. with their top corresponding reference genomes identified through BLAST analysis
